# Patient’s Perspective on Disease Burden, Remission Definition, and Symptoms Associated With Treatment Seeking: A Qualitative Study in Adult and Adolescent Patients With Crohn’s Disease

**DOI:** 10.1093/crocol/otaa033

**Published:** 2020-05-05

**Authors:** Helen Kitchen, Mallory Farrar, Tamara Al-zubeidi, Hannah Pegram, Theresa Hunter, April N Naegeli, Laure Delbecque, Vipul Jairath

**Affiliations:** 1 Clinical Outcomes Assessment, DRG Abacus, Manchester, UK; 2 Global Patient Outcomes-Real World Evidence, Eli Lilly and Company, Indianapolis, Indiana, USA; 3 Patient-Focused Outcomes Center of Expertise, S.A. Eli Lilly Benelux N.V., Brussels, Belgium; 4 Division of Gastroenterology, Western University & London Health Sciences Centre, London, Ontario, Canada

**Keywords:** Crohn’s disease, qualitative, disease burden, remission

## Abstract

**Background:**

Disease burden, a definition of remission, and symptoms that drive treatment seeking were explored in a Crohn’s disease (CD) population.

**Methods:**

A qualitative semistructured interview guide was developed, informed by published literature. Clinicians identified adolescents and adult patients with CD. Face-to-face interviews were audio-recorded and transcribed. Two rounds of interviews were conducted with patients. Transcripts were analyzed using thematic methods facilitated by ATLAS.ti.

**Results:**

Twenty-four patients participated in the first round of interviews (n = 16 adults, mean age 50.3 years; n = 8 adolescents, mean age 15.6 years). Abdominal pain (n = 24), urgent bowel movements (n = 24), diarrhea (n = 23), and frequent bowel movements (n = 21) were the most frequently reported symptoms. CD affected patients’ physical functioning, daily activities, emotional wellbeing, social functioning, work/education, and relationships. No major difference in disease burden was observed between adolescents and adults. Twenty-three patients (96%) reported they would seek or had sought medical treatment for at least one symptom including abdominal pain (n = 19), diarrhea (n = 12), and blood in stools/rectal bleeding (n = 9). On a 0–10 scale (0 = no symptom and 10 = symptom at its worst possible), most patients (87%, 20/23) answered they would seek/had sought treatment when the symptom’s severity was at least 7. In the second round of interviews (n = 6 adults, mean age 51.5 years), 5/6 patients described that they did not require a complete absence of abdominal pain or loose/watery stools to consider their CD to be in remission.

**Conclusions:**

CD is associated with substantial disease burden. Worsening of some symptoms drives treatment seeking. To some patients, remission is not defined as a complete absence of symptoms.

## INTRODUCTION

Crohn’s disease (CD) is a chronic inflammatory bowel disease of unknown etiology that can potentially affect any portion of the gastrointestinal (GI) tract from the mouth to the anus. It is characterized by periods of remission interspersed with intermittent flares that can lead to progressive bowel damage which can lead to intestinal resection.^1–3^ CD typically manifests between 15 and 30 years of age with symptoms of diarrhea, abdominal pain, weight loss, and rectal bleeding.^3^ This results in substantial impact on patients’ functioning, daily activities, wellbeing, and ability to work and is associated with economic and health-related quality of life (HRQoL) burden.^4–6^ Although the symptoms and impacts of diarrhea and abdominal pain are well documented and are routinely assessed in clinical trials and clinical practice,^4, 7, 8^ symptoms that drive both adult and adolescent patients to seek treatment are not well described from the patient’s perspective. Identification of the factors that drive treatment seeking is important because of the recognized high healthcare burden^6^ of CD. An understanding of such drivers may better inform healthcare resource use, aid identification of unmet treatment needs, and lead to improved patient outcomes. An understanding of what patients expect in order to consider a treatment to be successful is also required. Few studies have specifically explored patients’ perceptions of a successful treatment response and if/how they define CD remission.

In this study, we aimed to understand adult and adolescent patients’ experiences of CD, including CD-related symptoms, the burden of living with CD, as well as the symptoms that drive patients to seek medical treatment. Patients’ understanding and definitions of CD remission were also explored.

## MATERIALS AND METHODS

This study was designed as a cross-sectional, qualitative, noninterventional interview study. Two rounds of qualitative interview were conducted in patients with a confirmed diagnosis of CD.

### Patient Recruitment

#### Round 1

Patients were recruited from 2 sites in the United States by gastroenterologists from various private group medical practices that treat and manage patients with CD. The objectives of Round 1 were to understand (1) patients’ CD symptom experience, including and beyond abdominal pain and diarrhea, (2) the impact of CD on patients’ daily living, functioning, and wellbeing, and (3) symptoms that drive patients to seek medical treatment. Current symptom experience and symptoms reported during flares were explored. All patients were recruited according to predefined eligibility criteria. Gastroenterologists were required to complete and return case report forms to a specialist patient recruitment agency for all referred patients. Patients were eligible for inclusion if they were aged at least 12 years with a confirmed diagnosis of CD for at least 12 months and fluent in English. Patients were excluded if they had serious conditions other than CD that could impair their ability to participate in the study or affect the results of the study (eg, ulcerative colitis, abdominal abscesses, colonic mucosal dysplasia, intestinal obstruction, and irritable bowel syndrome) or had undergone recent abdominal surgery.

Diagnosis was based on a previous laboratory examination of blood and/or stool matter, X-ray, or endoscopic examination.

All clinical information was provided by referring clinicians (eg, disease severity, treatment regimens, and comorbidities). Purposive sampling is a nonrandom method of ensuring that particular characteristics within a sample are represented^9^ and was used to recruit a diverse and representative sample, including male and female patients, patients of non-white ethnicities, and patients educated to high school diploma or less.

#### Round 2

An additional sample of patients, who did not participate in Round 1, aged at least 18 years was recruited using the same referring strategy and eligibility criteria as in Round 1. The objective of these follow-on interviews was to explore adult patients’ understanding, perceptions, and definitions of disease remission; this was not a specific objective of the Round 1 interviews. Purposive sampling aimed to include patients who had experienced current or previous severe levels of CD as assessed by their clinician.

### Interview Procedure

#### Round 1 interviews

A semistructured interview guide was developed which was informed by a conceptual model (CM) of patient-reported symptoms and impacts of CD. The CM had been developed following a review of published qualitative literature.^4, 7, 10–19^ Patient-reported CD symptoms were split between 3 domains: “GI pain,” “GI symptoms,” and “other CD symptoms.” A number of symptoms that patients associated with CD were placed under “Extraintestinal manifestations.” Patient-reported impacts of living with CD were split between 3 domains: “GI-specific impacts,” “Impacts on wellbeing,” and “Overall impacts on HRQoL.” The CM and interview guide were reviewed by a clinician with expertise in the management of patients with CD to verify the clinical relevance and correct domain placement of the CM’s symptoms and impacts in addition to the appropriateness of the interview questions.

Patients participated in 90-minute face-to-face, one-to-one interviews conducted between December 9 and 16, 2018. A concept elicitation discussion included open-ended questions to explore patients’ experiences of CD, including the symptoms they experienced, and the associated impact of CD on their daily lives, functioning, and wellbeing. Next, patients were asked which of the symptom experiences that they had reported would drive/have driven them to seek medical treatment. Finally, patients were presented with a 0–10 scale (0 = no symptom and 10 = symptom at its worst possible) and asked to select a score at which they would seek medical treatment for each of those symptoms that they had reported during the discussion.

#### Round 2 interviews

The semistructured interview guide used in Round 1 was adapted to explore adult patients’ understanding and definitions of disease remission and expectations of treatment response. Patients participated in 30-minute telephone/WebEx interviews between February 25 and 27, 2019. An initial concept elicitation discussion involved open-ended questions to explore patients’ understanding and perceptions of CD remission. Additionally, patients were asked about their symptoms that would be present or absent during remission and asked to describe the severity, frequency, and extent of symptoms’ bother that would represent remission, with a particular focus on abdominal pain and total number of liquid/watery stool bowel movements (BMs).

All interviews (Round 1 and Round 2) were conducted by an experienced qualitative researcher and were audio-recorded and transcribed verbatim.

### Analysis

Demographic and clinical information collected during recruitment were descriptively summarized. Interview transcripts were analyzed using qualitative thematic techniques^20, 21^ aided by ATLAS.ti v7.5 software which facilitated the coding and organization of data. First, the transcripts were read by the lead analysts and overarching ideas were identified. Next, descriptive codes were assigned to quotes within the transcripts to summarize the core meaning of patients’ narratives, from which concepts and themes were identified. Codes were collated into potential themes and these themes were then compared and contrasted in order to interpret structure among them, both within patients and between patients according to clinical or demographic characteristics. Where new codes or themes emerged, the previous transcripts were reviewed to ensure that the code or theme was not missed during the first review. Through this method, a list of key concepts and themes were iteratively identified within each interview and across the sample. The first 2 transcripts were coded by several of the research team to develop a preliminary codebook for the interview. Using this codebook, the research analysts then coded the remaining interview transcripts.

Adult and adolescent analysis was initially conducted simultaneously as no prominent difference between the 2 groups was noted during the interviews. However, further subgroup analysis was conducted to identify any subtle differences.

Saturation analysis of CD symptoms reported by both the adult and adolescent groups was conducted following Round 1 (n = 24) interviews to determine if conceptual saturation was achieved. Conceptual saturation is defined as the point at which no new concept-relevant information emerges^22^ and is applied to spontaneously elicited concept elicitation data only. Patients transcripts were divided into 3 sets (n = 8) in chronological order. Set 1 was compared to Set 2 to identify any new symptom concepts that had arisen in Set 2. Similarly, Set 3 transcripts were compared with Set 1 and Set 2.

### Updated CM

The draft CM was updated following the Round 1 interviews (n = 24).

### Ethical Considerations

Ethical approval for this study was granted by the New England Independent Review Board (#120180264) on November 12, 2018. Informed consent was obtained from all of the adult patients and from the adolescents and their parent/guardian/caregiver.

## RESULTS

### Sample Characteristics

#### Round 1

Twenty-four patients participated in Round 1 of the interview study. Table 1 presents the sample clinical and demographic characteristics, summarized by adult and adolescent subgroups. Patients had been diagnosed with CD for a mean [range] years (adults 10.1 [1.0–23.7]; adolescents 2.8 [1.0–7.7]) and both adult and adolescent groups contained an equal proportion of male and female patients. The majority of patients were Caucasian or white (n = 16, 67%). Eighteen (75%) patients had moderate or severe CD as rated by the referring clinician and 18 (75%) patients received treatment for their CD; 17 (71%) were in receipt of a biologic therapy. Two patients were prescribed corticosteroids.

**TABLE 1.** Round 1 Sample Clinical and Demographic Characteristics

Clinical/Demographic Characteristic	Adult Patients (N = 16), n (%)	Adolescent Patients (N = 8), n (%)	Total (N = 24), n (%)
Sex			
Male	8 (50%)	4 (50%)	12 (50%)
Female	8 (50%)	4 (50%)	12 (50%)
Age, years			
Mean (median) [range]	50.3 (51.5) [27.0–75.0]	15.6 (15.5) [14.0–17.0]	38.7(45.5) [14.0–75.0]
Ethnicity			
Caucasian or white	11 (69%)	5 (63%)	16 (67%)
Black or African American	3 (19%)	1 (13%)	4 (17%)
Hispanic	2 (13%)	2 (25%)	4 (17%)
Education (highest level completed)			
High school diploma or equivalent	5 (31%)*	—	—
College or associate’s degree	5 (31%)	—	—
Bachelor’s degree	4 (25%)	—	—
Graduate degree	1 (6%)	—	—
Other	1 (6%)	—	—
Time since diagnosis, years			
Mean (median) [range]	10.1 (10.6) [1.0−23.7]	2.8 (1.4) [1.0−7.7]	7.6 (7.0) [1.0−23.7]
Disease severity (reported by referring clinicians)			
Mild	2 (13%)	4 (50%)	6 (25%)
Moderate	13 (81%)	3 (38%)	16 (67%)
Severe	1 (6%)	1 (13%)	2 (8%)
Comorbidities			
High blood pressure	3 (19%)	0 (0%)	3 (13%)
Diabetes mellitus	1 (6%)	0 (0%)	1 (4%)
Treatment (current)^†^			
Adalimumab (HUMIRA, AbbVie, North Chicago, IL)	8 (50%)	2 (25%)	10 (42%)
Vedolizumab (ENTYVIO, Takeda US, Deerfield, IL)	3 (19%)	0 (0%)	3 (13%)
Infliximab (REMICADE, Janssen, Titusville, NJ)	2 (13%)	2 (25%)	4 (17%)
Prednisone/corticosteroids^‡^	2 (13%)	0 (0%)	2 (8%)
No treatment (including modified diet/probiotics)	2 (13%)	4 (50%)	6 (25%)
Time on biologic treatment, mean [range]	N = 13, 4.4 [0.3–16.9]	N = 4, 0.8 [0.2–2.3]	—
Time since diagnosis years, mean [range]	11.5 [1–17.25]	1.43 [1.08–2.3]	—

*n = 1 completed some college.

^†^Other treatments: n = 1 ciprofloxacin every 5 days and Imodium A-D as required, n = 1 azathioprine (IMURAN, Aspen Pharma Trading Ltd, Dublin, Ireland) 5 mg/day.

^‡^Patients prescribed corticosteroids also received Adalimumab (HUMIRA^®^, AbbVie, North Chicago, Illinois).

#### Round 2

Six adult patients participated in Round 2 of the interview study. The sample included male (n = 2) and female (n = 4) patients with a mean age of 51.5 years (range 41–74). Most patients (83%; n = 5) were Caucasian or white and 1 patient was Hispanic. Most patients (66%; n = 4) were educated to a graduate level. Patients had been diagnosed with CD for a mean 7.1 years (range 1–17). Three (50%) of the patients had severe CD in the opinion of their clinician while the rest of the sample (50%) had moderate CD. All 6 patients received biologic therapy and 3 (50%) were also prescribed corticosteroids.

## ROUND 1 INTERVIEWS (N = 24)

### Disease Burden

#### CD symptoms experienced by patients

Twenty-five symptoms were reported by patients in Round 1 of the interview study. Symptoms reported by more than 1 patient are presented in Table 2 by adolescent and adult subgroups. Symptoms that were reported spontaneously or reported following interview probing techniques are noted.

**TABLE 2.** Symptoms Reported by Patients in Round 1

	Reported (N = 24)			
Symptom	Adult total, N = 16	Adolescent Total, N = 8	Overall Total, n (%)	Example Quotes
*GI pain*				
Abdominal pain	16 (15s; 1p)	8 (7s; 1p)	24 (100%)	“So, I wake up and I’ll usually wake up because of stomach pain, and then, I’ll go to the bathroom.” (01-07-P)
Abdominal cramps	10 (9s; 1p)	4 (4s; 0p)	14 (58%)	“The stomach cramps and the pain, it was really, really tender.” (01-11- A)
Rectum pain/soreness	4 (3s; 1p)	2 (1s;1p)	6 (25%)	“Your anus hurts. Everything hurts.” (02-02-A)
*GI symptoms*				
Diarrhea	16 (14s; 2p)	7 (3s; 4p)	23 (96%)	“I have flare-ups or bouts of diarrhea and that kind of thing.” (01-13-A)
Bowel movement urgency	16 (10s; 6p)	8 (2s; 6p)	24 (100%)	“There are times I have to run to the bathroom with diarrhea. Like this morning, I did, which is why I was almost late, but I wasn’t late.” (02-11-A)
Frequent bowel movements	15 (8s; 7p)	6 (3s; 3p)	21 (88%)	“Before my treatment started it was pretty rough, ten to 15 times to the bathroom a day.” (01-12-A)
Blood in bowel movement/ rectal bleeding	10 (10s; 0p)	1 (0s;1p)	11 (46%)	“When we really found out that I had Crohn’s, I just had an episode, when I went to the restroom, and I had a lot of bleeding and I passed out.” (01-11-A)
Bloating	7 (7s; 0p)	4 (4s;0p)	11 (46%)	“I guess it’s just the bloating and the pain, the discomfort.” (02-06-A)
Nausea	6 (6s; 0p)	3 (3s; 0p)	9 (38%)	“I get nausea, and when I’m having flare-ups and my nausea’s a lot worse.” (01-08-A)
Vomiting	4 (4s; 0p)	2 (2s; 0p)	6 (25%)	“I’ve been diagnosed for a little over a year, and before I was even diagnosed, I had a lot of symptoms, I would throw up a lot.” (01-07-P)
Heartburn/ indigestion	2 (2s; 0p)	2 (2s; 0p)	4 (17%)	“I’ll get heartburn and then I’ll vomit, and it just—it takes a good couple of hours from my day ‘cause I’ll just be sitting in the bathroom vomiting’.” (01-07-P)
Gas	2 (2s; 0p)	1 (1s; 0p)	3 (13%)	“It’s just constant bathroom breaks. And stomach pains, gas, bloating.” (01-07-P)
Constipation	2 (2s; 0p)	0 (-)	2 (8%)	“One thing about Crohn’s disease, you can have constipation versus diarrhea.” (02-02-A)
Gurgling/noises	1 (1s; 0p)	1 (1s; 0p)	2 (8%)	“It’s just pressure and gurgling, the noises, I guess…] if my stomach is being loud, I know that pain is coming ‘cause it’s just… tumultuous, I guess, wild’.” (01-07-P)
*Other CD symptoms*				
Fatigue/tiredness	8 (6s; 2p)	4 (4s; 0p)	12 (50%)	“I could get a good ten hours of sleep at night and wake up exhausted…Go to school and fall asleep in class.” (01-07-P)
Weakness	5 (5s; 0p)	0 (-)	5 (21%)	“I’m going to, kind of like, lay around trying to keep my strength up, you know and everything. I’m trying to explain to her, my, my son and wife, that, ‘You don’t understand’ you know, it’s that sometime that it just zaps the strength.” (01-10-A)
Weight loss	3 (3s; 0p)	0 (-)	3 (13%)	“I had a lot of weight loss because I didn’t have any appetite, so I lost weight. [… It affected me] actually in a good way, I was too fat.” (01- 10-A)
Lack of energy	2 (2s; 0p)	1 (1s; 0p)	3 (13%)	“I find that my energy level really drops…I have to be very careful of when my energy is high and low, especially after meals.” (02-01-P)
Fever	2 (2s; 0p)	1 (1s; 0p)	3 (13%)	“Sometimes I would have some nausea and a fever, or elevated temperature that would go along with it.” (02-03-A)
Back discomfort/ pain	2 (2s; 0p)	0 (-)	2 (8%)	“I feel pretty good so far, except for back pain.” (02-12-A)

CD, Crohn’s disease; GI, gastrointestinal; p, probed; s, spontaneously reported.

Abdominal pain/cramps and GI symptoms, including urgent BMs, diarrhea, frequent BMs, blood in BMs/rectal bleeding, bloating, and nausea/vomiting, were the symptoms most frequently reported by the patients.

All 24 patients (n = 16 adults, n = 8 adolescents) reported experiencing abdominal pain and urgent BMs. The severity of abdominal pain varied and patients described the most severe pain as occurring during a flare. The frequency at which patient experienced abdominal pain also varied; some patients experienced abdominal pain 2 to 3 times a day or every other day, while others only experienced abdominal pain in the context of less frequent flares which occurred every 2 to 3 months.

Most patients described urgency to have a BM as an immediate need to go to the toilet. Frequency of urgency was different among patients, with some patients experiencing urgency every day. However, some patients reported experiencing urgency only a few times a month. Severity of urgency also varied among patients, with some patients describing instances when they found it hard to “hold in” their BMs. Many patients also reported that urgency of BM was not specifically related to one type of BM such as diarrhea; patients reported that food could trigger urgent BMs and this would often be experienced at the same time as abdominal pain and cramping.

Additionally, the majority of patients had experienced diarrhea (96%; n = 16 adults, n = 7 adolescents) and frequent BMs (88%; n = 15 adults, n = 6 adolescents).

Patients described diarrhea as loose or liquid BMs, with the frequency and consistency of BMs varying between patients. Some patients experienced diarrhea every week, while other patients experienced diarrhea occasionally over the course of a few months. Diarrhea could co-occur with abdominal pain, abdominal cramping, and blood in the stool.

The frequency of BMs varied between patients, but all patients acknowledged experiencing an increased frequency of bowel moments during a flare and following abdominal pain, cramping, and discomfort. Several patients felt that eating certain foods could increase the frequency of their BMs.

Other non-GI symptoms that were frequently reported by patients included fatigue/tiredness (50%; n = 8 adults; n = 4 adolescents) and weakness (21%; n = 5 adults).

Symptoms that were reported by one patient each included peri-anal discharge, leg pain, back pain, dizziness, and shortness of breath. Extraintestinal manifestations such as joint pain, hair loss, skin and eye problems were also reported by a small number of patients.

**FIGURE 1.**
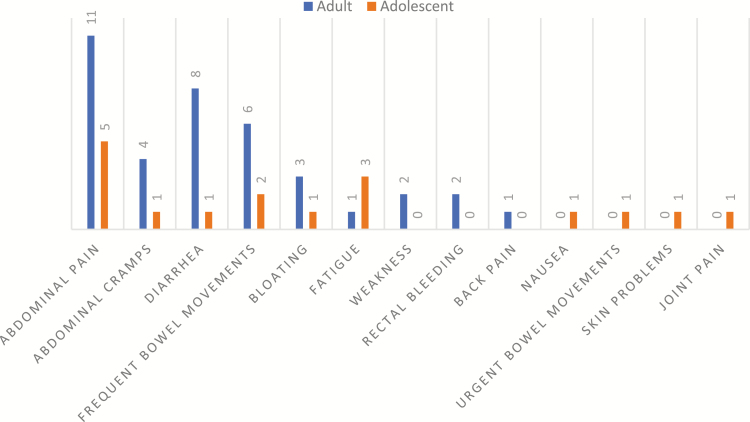
Patients’ most bothersome symptoms reported in Round 1 interviews (N = 24)^†^. ^†^Counts do not equal 24 as n = 18 patients reported more than one bothersome symptoms.

All 24 patients were asked which of their CD symptoms bothered them the most. Figure 1 summarizes the responses. Most patients (75%; n = 13 adults, n = 5 adolescents) reported 2 or 3 symptoms that they considered to be the most bothersome. Abdominal pain, abdominal cramping, and GI symptoms (diarrhea, frequent BMs, bloating, rectal bleeding, nausea, and urgent BMs) were predominantly the most bothersome for patients: abdominal pain (66%; n = 11 adults, n = 5 adolescents), abdominal cramping (21%; n = 4 adults, n = 1 adolescent), diarrhea (38%; n = 8 adults, n = 1 adolescent), and frequent BMs (33%; n = 6 adults, n = 2 adolescents), for example, “The abdominal pain, the cramping and the diarrhea, the frequency that I have to go to the bathroom” (02-11-A). Non-GI symptoms that were among the most bothersome included fatigue (17%; n = 1 adult, n = 3 adolescents) and feeling weak (8%, n = 2 adults).

Abdominal pain appeared to be almost equally bothersome for both adult and adolescent patients, while diarrhea appeared to be more bothersome for adults than for adolescents even though diarrhea was reported in similar proportions among both groups (n = 16/16 adults; 7/8 adolescents). Interestingly most adolescent patients who described experiencing fatigue/tiredness (3/4) also reported it as one of their most bothersome symptoms, compared with only 1/8 adult patients who had experienced fatigue/tiredness.

#### Flares

The concept of “flares” was discussed with patients; most (92%; n = 22) were familiar with and used the term flares or flare-ups to describe times when their usual symptoms (eg, abdominal pain, frequent BMs) substantially worsened in severity or times when symptoms that were not usually present occurred (eg, nausea, vomiting, and blood in stools). Only 2 of the adolescent patients were unfamiliar with the term “flares.”

All 24 patients reported having experienced worsening and/or the reoccurrence of one or more of their symptoms during the course of their CD, for example, “Just like all of those symptoms really worse, if they’re at their worst and maybe needing to see the Doctor or get another medication for that time” (02-04-A), “When I have a flare-up I get some, sometimes diarrhea, very uncomfortable. I: *So, could you tell me the difference. in a day when you’re flaring and a day when you’re not flaring?* I feel okay…I don’t have any symptoms” (02-14-A).

The most frequently reported flare symptoms were abdominal pain/cramping (83%; n = 20) and changes in BMs, including more frequent BMs and/or diarrhea (71%; n = 17), for example, “I’d say, like, a flare-up is, like, your most severe symptoms, like cramping and bloating, all at once and you have, like, an urgency and it lasts pretty long” (02-05-P). In general, there were no major differences reported between adult and adolescent flare experiences.

#### Impacts of living with CD

All patients in Round 1 of the interview study reported impacts they had experienced as a consequence of living with CD. CD affected patients’ daily activities and ability to fully participate in their life activities.

#### Impacts on physical functioning and daily activities

The most prominent impact was on diet and eating. Most patients (n = 15 adults, n = 6 adolescents) described the need to change their diet and avoid certain foods to prevent triggering symptoms such as pain, cramping, urgent BMs, diarrhea, and frequent BMs.

Patients’ physical functioning was affected by pain. Adolescent patients may have been more affected due to scheduled school physical activities which they sometimes could not participate in, for example, “I played lacrosse and after I got sick it was a lot harder to move around. Like, if—I was going to go back on the swim team this year…but I couldn’t because with what I, what I usually swam, it would cause me very, very bad pain in my, my abdominal area” (01-06-P).

#### GI-specific impacts

Impacts that were specific to GI symptoms included the need to go to the toilet frequently (n = 13 adults, n = 7 adolescents), dealing with unpredictable GI pain, urgent BMs, and vomiting (n = 9 adults, n = 3 adolescents), for example, “I’m asking two or three, four times, to go to the bathroom in one period [lesson] by, by myself” (01-05-P).

Patients were constantly seeking the nearest toilet (n = 6 adults, n = 2 adolescents), for example, “… always checking wherever I am in unfamiliar places, where the bathroom is and how long it takes to get there” (02-01-A) and were also worried about not reaching the toilet in time due to their urgent need to have a BM (n = 7 adults), for example, “I’m worried that I might even go in my pants” (02-13-A).

#### Impacts on social functioning, hobbies, and leisure pursuits

Sixteen adult and 5 adolescent patients reported that their social lives had been affected due to CD. The unpredictable nature of symptoms made it difficult for patients to make plans as they could not be sure they would be well enough to attend, for example, “In some ways I, kind of, socially pull back” (02-01-A).

#### Impacts on work and education

Twenty patients (n = 13 adults, n = 7 adolescents) reported that CD had impacted their work or school life. Most patients (n = 7 adults, n = 6 adolescents) had needed to miss days at work or school due to abdominal pain and BMs, for example, “Like about a month before for work and then recently, I was out, like, a week, well, every month. I miss work probably about two or three days” (01-01-A).

#### Impacts on emotional and psychological wellbeing

Many patients (n = 13 adults, n = 2 adolescents) were anxious about urgent BMs, locating and getting to a bathroom in time, experiencing flares, and the future implications of having CD.

Patients also reported feeling embarrassed and/or self-conscious (n = 10 adults; n = 4 adolescents), especially with relation to frequent bathroom use, for example, “Kind of embarrassing when you’re at school, you know? You don’t really tell people that. You just have to tell the Teacher… ‘I have an emergency, I got to use the bathroom’” (02-08-P). Feelings of sadness, upset, and depression were often described by patients (n = 9 adults, n = 5 adolescents), either as a generic feeling accompanying their experience of CD symptoms or more specifically when they reflected on the impact of CD on their work, family, and social life, for example, “It does affect me on a daily basis… it’s not only physically, it’s mentally you get a little bit down, because, …you’re incapacitated” (02-11-A).

#### Impacts on relationships

Relationships, including friendships, were affected by CD; adolescents, in particular, described feeling left out of social situations and losing friends due to being unable to participate in activities, for example, “I haven’t been on any sports teams, so I’ve lost a couple of friends through that” (01-06-P).

### Updated CM

The findings from this study, specifically the concepts arising from the open-ended discussion in Round 1, were compared to the CM developed from the literature review to provide support for existing concepts and identify any potential new concepts relating to CD symptoms and impacts.

**FIGURE 2.**
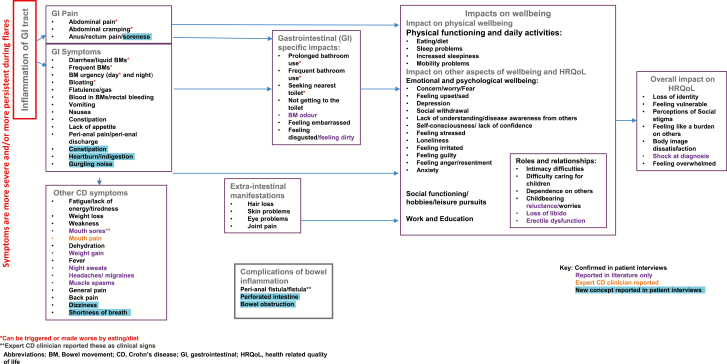
Updated conceptual model following Round 1 interviews (n = 24).

Most symptom and impact concepts within the CM were also identified in this study. At a domain level, GI pain symptoms, GI symptoms, and other CD symptoms were all reported by patients within this study. Three new GI symptoms arose as follows: constipation, heartburn/ indigestion, and gurgling noises. In addition, 2 new concepts unrelated to pain or GI symptoms also arose as follows: dizziness and shortness of breath. No new impact concepts were identified from the interviews. Figure 2 represents the updated CM.

### Treatment Seeking

Twenty-three patients (96%; n = 16 adults, n = 7 adolescents) confirmed that they would seek or have sought medical treatment for at least one of their symptoms. Seventeen (n = 13 adults, n = 4 adolescents) reported that they have sought or would seek help for more than one symptom. Patients stated that they would either call or go to their primary/specialist doctor or attend a hospital emergency room (ER).

Only one adolescent patient reported that they would not seek additional medical treatment for their symptoms. This may be due to their mild disease severity as confirmed by their clinician. This patient reported that they were able to manage their symptoms by diet, rest, and nonprescription pain relief as needed.

Abdominal pain was the most frequently reported symptom that patients would seek medical treatment for (n = 14 adults, n = 5 adolescents). Patients described constant or prolonged “uncontrollable” pain that could not be alleviated by nonprescription medication that rendered them immobile and “doubled-up,” “on the floor,” or “in tears” from the pain.

Diarrhea (n = 10 adults, n = 2 adolescents) that was either constant, frequent, occurred both during the day and night, not controlled by nonprescription medication or that was accompanied by abdominal pain or nausea were all cited as reasons for seeking treatment. Dehydration due to constant diarrhea was a concern for a few patients.

Blood in stools/rectal bleeding was reported by 9 adults. Most patients would only seek treatment if they experienced what they considered large amounts of blood seen when using the toilet.

Fatigue/tiredness (n = 1 adult, n = 3 adolescents) that was described as “excessive” or “unbearable” and that caused excessive sleepiness during the day despite adequate nighttime sleep was also a driver of treatment seeking.

Bloating (n = 2 adults, n = 1 adolescent) either on its own or accompanied by diarrhea or severe abdominal pain, “forceful” vomiting/constant nausea (n = 1 adult, n = 2 adolescents) and fever (n = 1 adult, n = 1 adolescent), severe rectal pain (n = 1 adolescent), and severe joint pain (n = 1 adolescent) were also cited as reasons to seek treatment.

Nine patients (n = 8 adults, n = 1 adolescent) explained that they would seek medical help, for example, attend hospital or go to their doctor, if they experienced a flare-up or a combination of symptoms or developed new symptoms.

**FIGURE 3.**
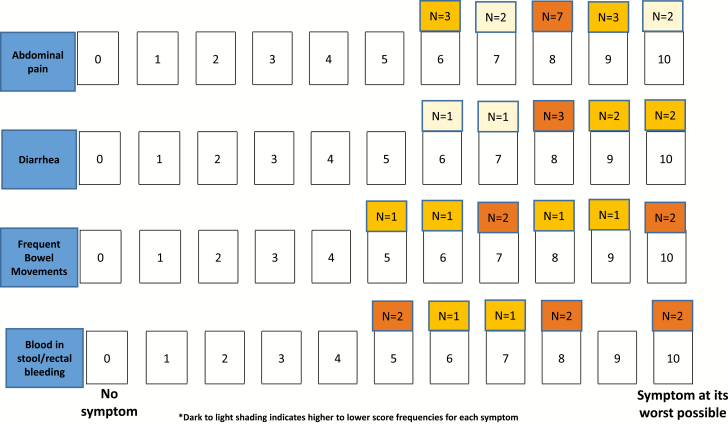
Patients’ numerical rating scale (NRS) scores for seeking medical treatment (n = 22)^†,‡^. ^†^Seven patients selected adjacent scores (eg, 6 or 7); only the lower score is reported in the figure. ^‡^These symptoms were reported by some patients during the general discussion but were not additionally discussed during the NRS task: frequent bowel movements (n = 4), abdominal pain (n = 3), and diarrhea (n = 2).

Figure 3 represents the 4 most frequently reported symptoms that have driven or would drive patients to seek medical treatment. Most patients (20/23) reported that they would seek/had sought treatment when the symptom’s severity was at least 7 on a 0–10 numerical rating scale. The severity score ranges that would prompt patients to seek medical treatment for diarrhea and abdominal pain were 6–10. Patients reported a range of 5–10 for frequent BMs and blood in stools/rectal bleeding.

A few patients explained they were pain-tolerant therefore selected higher scores for abdominal pain, while a few other patients selected lower scores for symptoms such as constant diarrhea or blood in stools/rectal bleeding than their other symptoms, as they were considered more disruptive, important, or more worrisome.

## ROUND 2 INTERVIEWS (N = 6)

### Remission Definition

#### Patient terminology

All 6 adult patients in Round 2 of the interview study were asked if they had ever heard the term “remission” in relation to their CD and what they called “remission” or times when their CD had “got better.” Three patients had heard this term in relation to CD, however, one of these patients had never used the term “remission” in relation to an improvement in their CD symptoms. Two patients had not heard this term used in relation to CD and another patient had only heard the term used in relation to cancer. The patients who had heard the term “remission” in relation to CD (or another condition) had graduate-level education whereas the patients who had not heard this term had lower educational attainment.

#### Definition and experiences/perceptions of remission

The 3 patients who had heard the term “remission” in relation to CD were asked how they would define “remission” in CD. All 3 considered symptoms that were “not severe” or had “eased” to define remission, for example, “It’s when your symptoms are not as severe, and you’re in a calm spot” (02-17).

The 3 patients who had not heard the term remission previously in relation to CD were asked how they would define times when their CD had got better (n = 3). All 3 cited a reduction in abdominal pain, diarrhea, and frequency of BMs as defining the times when their CD had got better, for example, “When I’m not experiencing pains in my stomach and probably not going to the bathroom quite as often” (02-19). One patient also cited fewer bathroom visits (BMs) and one cited a lack of fatigue. One patient felt that they never had experienced remission because their symptoms always returned. This patient associated remission with a permanent absence of symptoms, for example, “I consider remission where they’re permanently gone or they’re not going to come back, and I’ve never been through that, so I can’t really consider myself in remission or anything” (02-16).

All 6 patients were asked if all of their CD symptoms would have to go away for them to consider themselves to be in “remission.” Five patients reported that not all their symptoms would need to go away to consider themselves in remission, for example, “I just wanted just a drastic improvement. And, you know, and just issues, maybe, few and far between” (02-18). Only one patient required that all of their symptoms, including nausea, vomiting, fatigue, and urgent BMs, would have to go away to consider themselves in remission.

Six patients confirmed that both the number of liquid/watery BMs and the severity of their abdominal pain needed to improve to consider their CD in “remission,” for example, “I think they go together, they’re part and parcel… whenever you have diarrhoea you have discomfort … the less I have to deal with the pain, the less I have to go to the bathroom… the better it makes my life” (02-19).

All 6 patients were asked the number of liquid/watery (type 6/7) BMs they would have in a typical 24-hour period to consider their CD in remission. Additionally patients were asked to specify the smallest number of liquid/watery (type 6/7) BMs that was acceptable for them to consider themselves to be in remission. The number of BMs ranged from 0 to 5 and 0 to 4, respectively (Table 3). Patients were also asked which level of abdominal pain would indicate CD remission. Most patients identified “mild” as the level of abdominal pain that would indicate CD remission (Table 4).

**TABLE 3.** Round 2 Patients’ Perceptions of Remission: Liquid/Watery BMs

Patient ID	Number of Liquid/Watery BMs in Typical 24 h which Patients Perceive as CD Remission	Smallest Number of Liquid/Watery BMs in Typical 24 h which Patients Perceive as CD Remission
Pt-01	0	0
Pt-02	2	1
Pt-03	0–2	2
Pt-04	0–1	1
Pt-05	4–5	4
Pt-06	4–5	3

BM, bowel movements; CD, Crohn’s disease.

**TABLE 4.** Round 2 Patients’ Perceptions of Remission: Abdominal Pain

Level of Abdominal Pain That Represents Remission	n (%) (N = 6)
None	1
Mild	4
Moderate	1

## SATURATION ANALYSIS

Saturation analysis showed that almost all symptom concepts were identified in the first 2 sets of the Round 1 interviews. Only 2 new concepts emerged in Set 3: leg pain and back pain/discomfort. However, “general pain” in multiple locations was reported in previous sets; therefore, saturation was considered achieved. Saturation analysis was not conducted for the Round 2 interviews due to the difference in study objectives, the exploratory nature of this second round of interviews, and the small sample size of 6 patients.

## DISCUSSION

Several symptoms and impacts on wellbeing were described by the patients in this qualitative study which demonstrated the substantial disease burden that adult and adolescent patients with CD are faced with. Most of the symptoms and impacts reported by patients were also identified in the targeted literature review conducted before the interviews as shown in the CM (Fig. 3). The symptoms and impacts reported in this study are in-line with those included in the CM developed by Gater et al.^23^

Abdominal pain/cramps and GI symptoms including diarrhea, frequent BMs, bloating, and blood in BMs were the most frequently reported and most bothersome symptoms for patients. This result is in-line with other qualitative studies that identified these symptoms as common and important symptoms experienced by patients with CD.^7, 12, 14^ There were few differences between symptom reports from males and females although notably more females than males reported bloating. Urgent BMs were also notably problematic for those who experienced them because they were a cause of substantial anxiety. Patients always had to know where the closest toilet was and feared losing control of their BMs. This finding is also noted in other CD studies.^14, 19^ The non-GI symptoms which were considered bothersome included fatigue, weakness, and joint pain; indeed, joint pain may be one of the most severe and difficult symptoms for patients with CD to manage.^7, 12^

This study provided an insight into patients’ experience of flares which were found to be disruptive to patients’ daily lives and functioning; a number of patients experienced variation in the severity of their flares which has been noted in previous research.^11^ Several patients sought additional medical help when they considered themselves to be having a flare, including going to their primary care doctors, seeking different medication or even visiting the ER.

A small sample of adolescent patients were included in this study and few differences in the type of symptoms reported were identified between adults and adolescents. The impact of frequent BMs appeared to be slightly more prominent in adolescents, mainly due to the disruption of lessons at school. Additionally, nearly half of the adolescent sample cited fatigue as one of their most bothersome symptoms and also reported that they would seek treatment for their fatigue. However, comparisons based on this small sample should be considered exploratory only. Indeed, there appears to be a paucity of published qualitative studies that explore the lived experiences of adolescents living with CD, despite the common incidence and increasing prevalence of CD in adolescents.^24^

Worsening of the symptoms that generally characterize CD, for example, abdominal pain, frequent BMs, and diarrhea were the main drivers of treatment seeking among patients. Interestingly, when patients do seek treatment they do not necessarily expect all of their symptoms to be completely alleviated. Instead a reduction in severity in abdominal pain and loose/watery stools may be accepted as a satisfactory treatment outcome. This finding potentially reflects patients’ expectations for living with a chronic condition; Norton et al^7^ found that patients did not expect to regain their prediagnosis health status and many reported being in remission or feeling good despite still experiencing CD symptoms. However, other studies have noted that some patients defined remission as no symptoms present and not requiring steroid treatment.^25^ Further exploration of patient perceptions of CD remission and their perceptions of treatment expectations would help align patient and healthcare professional treatment outcome goals and aid the evaluation of treatment success in patient-focused drug development.

Limitations of this study are acknowledged. This study was completed in the United States only with English-speaking patients and therefore the results may not be applicable to other countries and cultures without further investigation. While the sample in Round 1 included adolescent patients, no younger adolescents aged 12–13 years were included. However, diagnosis of CD in American patients is most common between the ages of 15 and 35^26^ and therefore the lack of participation among 12- to 13-year olds is reflective of the population. Additionally, the sample overall included patients with a age range of 14–75 years who shared their experiences. Subgroup analyses to identify differences and similarities between adult and adolescent patients were somewhat limited by the sample size and therefore any differences highlighted in the data should be treated with caution. The Round 2 interviews included only short discussions with a small sample of adult patients who were mostly educated to graduate level and therefore the findings should be considered exploratory; further confirmation in a larger and more diverse sample is recommended. There is an inherent risk of sampling bias in that those who were interested in participating in the study may be those who are more likely to seek care to begin with. In addition, no objective disease severity/activity assessment was collected at recruitment. Finally, qualitative data interpretation can be influenced by the interviewers’ and analysts’ personal biases. This was somewhat mitigated by using the same interviewer in all interviews so that interviewer effects were consistent. Additionally, the findings of this study were reviewed by a multidisciplinary team including clinical experts.

## CONCLUSIONS

This qualitative study explored the lived experience and disease burden of CD which was primarily associated with GI symptoms and abdominal pain which significantly impacted patients’ daily activities, physical functioning, sleep, work/school, and emotional wellbeing. Treatment seeking was generally driven by worsening in severity of GI symptoms, while CD disease remission was primarily considered by patients to be associated with an improvement or reduction in symptoms including abdominal pain and BMs.

## Data Availability

The data that support the findings of this study are available on reasonable request from Eli Lilly [April Naegeli]. The data are not publicly available due to protect the privacy of research participants.

## Notes

Funding: The study funding was provided by Eli Lilly & Co.
